# Terahertz Active Photonic Crystals for Condensed Gas Sensing

**DOI:** 10.3390/s110606003

**Published:** 2011-06-03

**Authors:** Alexander Benz, Christoph Deutsch, Martin Brandstetter, Aaron M. Andrews, Pavel Klang, Hermann Detz, Werner Schrenk, Gottfried Strasser, Karl Unterrainer

**Affiliations:** 1 Photonics Institute and Center for Micro- and Nanostructures, Vienna University of Technology, Gusshausstrasse 29/387, Vienna, Austria; E-Mails: christoph.deutsch@tuwien.ac.at (C.D.); martin.brandstetter@tuwien.ac.at (M.B.); karl.unterrainer@tuwien.ac.at (K.U.); 2 Institute of Solid-State Electronics and Center for Micro- and Nanostructures, Vienna University of Technology, Floragasse 7/362, Vienna, Austria; E-Mails: aaron.andrews@tuwien.ac.at (A.M.A.); pavel.klang@tuwien.ac.at (P.K.); hermann.detz@tuwien.ac.at (H.D.); werner.schrenk@tuwien.ac.at (W.S.); gottfried.strasser@tuwien.ac.at (G.S.)

**Keywords:** terahertz, optical microsensor, quantum-cascade laser, active resonator

## Abstract

The terahertz (THz) spectral region, covering frequencies from 1 to 10 THz, is highly interesting for chemical sensing. The energy of rotational and vibrational transitions of molecules lies within this frequency range. Therefore, chemical fingerprints can be derived, allowing for a simple detection scheme. Here, we present an optical sensor based on active photonic crystals (PhCs), *i.e.*, the pillars are fabricated directly from an active THz quantum-cascade laser medium. The individual pillars are pumped electrically leading to laser emission at cryogenic temperatures. There is no need to couple light into the resonant structure because the PhC itself is used as the light source. An injected gas changes the resonance condition of the PhC and thereby the laser emission frequency. We achieve an experimental frequency shift of 10^−3^ times the center lasing frequency. The minimum detectable refractive index change is 1.6 × 10^−5^ RIU.

## 1. Introduction

Optical microsensors based on high-Q cavities are very attractive for a wide range of chemical, biomedical or environmental applications. They offer high sensitivity, fast system response, low detection limit and simple fabrication. Different types of microresonators are used: cylinders [1], rings [2], toroids [3], spheres [4] and photonic crystals (PhCs) [5]. The mentioned resonators except for PhCs confine the optical mode by total internal reflection; whispering gallery modes are created. In PhC cavities, the mode is confined by multiple Bragg reflections. Both mechanisms ensure a strong modal confinement and a high-Q factor of the resonator.

These microcavities are used as local sensors. The analyte is brought into close distance to the resonator. More precisely, it is brought into the evanescent field of the cavity mode. Thereby, the optical properties of the resonator are altered. Two different schemes are used for the optical read-out. If the analyte shows an absorption in the frequency range of interest, the imaginary part of the refractive index and thereby the width of the resonance is varied [6]. More frequently, changes in the real part of the refractive index are sensed via shifts in the position of the resonance [6]. This explains also the use of high-Q cavities for sensing. The resonator linewidth determines the detection limit, as it is the smallest detectable frequency shift due to changes in the refractive index [7].

The standard detection scheme typically uses a passive, high-Q resonator. One way to improve the sensor’s performance are active cavities. Thereby, optical gain is directly included into the resonator which reduces the linewidth and thereby improves the sensitivity and the detection limit [8]. This is done typically by incorporating fluorescent dyes [4] or quantum dots [9] into the resonator. These gain media work excellently in the visible or near-infrared part of the spectrum. The same detection scheme can be extended to the mid-infrared and terahertz (THz) part of the electromagnetic spectrum. Different molecules show a characteristic absorption in these frequency regimes, they can be identified easily by their *chemical fingerprint*. Details on the THz range are discussed in Section 2.

Here, we present an active PhC microsensor operating in the THz spectral regime. The active PhC acts as an electrically pumped semiconductor laser operating at cryogenic temperature [10]. Therefore, no external light source has to be coupled into the cavity. The emission frequency is a function of the gas concentration. As the gain medium, we use an active THz quantum-cascade layer sequence, which is incorporated directly into the PhC cavity. The size of the resonator is on the order a few hundred micrometers, which is comparable to ultra-high-Q microsensors in the visible [3,11]. However, the wavelength is two orders of magnitude larger than in the visible, which dramatically reduces the ratio of modal volume and effective wavelength. Our resonators achieve a ratio $V_{\text{res}}/\lambda_{\text{eff}}^{3}$ of 7.5, where *V_res_* is the total volume of the resonator and *λ_eff_* the effective wavelength in the active medium. If the mode distribution is taken into account to calculate the true modal volume *V_modal_*, the ration $V_{\text{modal}}/\lambda_{\text{eff}}^{3}$ is reduced to 2.5. Details on the THz gain medium are given in Section 2, and the device is described in Section 3.

## 2. Working in the Terahertz Spectral Region

**Figure 1.**
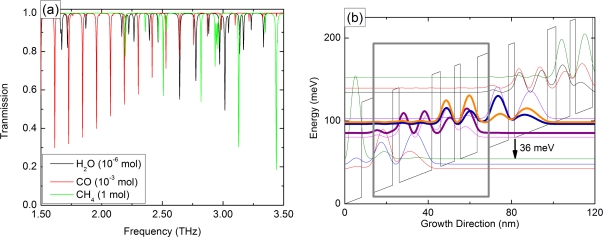
**(a)** Transmission of water vapor, methane, carbon monoxide at room temperature and 1 bar [16]. The individual absorption lines are clearly visible. **(b)** Calculated bandstructure of the THz-QCL. One cascade is marked with the gray box. The growth sequence in nanometers is 9.2/**3.0**/15.5/**4.1**/6.6/**2.7**/8.0/**5.5**, where the Al._15_Ga._85_As barriers are marked with bold letters. The lower lasing state is depopulated by a resonant longitudinal optical phonon.

The THz spectral region covers frequencies from 1 to 10 THz or, in terms of photon energy, 4 to 40 meV. It is a chemical fingerprinting region, making it highly interesting for sensors. The energy of rotational and vibrational transitions of molecules lies within this frequency range, leading to characteristic resonances. Therefore, it is easy to distinguish different molecules by their optical absorption. A transmission spectrum of methane, water vapor and carbon-monoxide is presented in Figure 1(a). The different absorption lines for each molecule are clearly visible. The same scheme can also be used to detect liquids [12] and solids, e.g., explosives [13,14]. Furthermore, THz radiation is not ionizing due to the low photon energy. Medical diagnostics is therefore showing a growing interest in the THz range, e.g., cancerous tissue can be identified [15].

For the realization of sensors, the THz frequency range offers also a few practical advantages. The mentioned frequency range of 1 to 10 THz corresponds to a free space wavelength of 300 to 30 *μ*m. Therefore, the fabrication of high-Q resonators becomes very easy. It is only necessary to ensure a roughness which is small compared to the wavelength. Standard optical lithography and reactive ion etching results in smooth structures in the THz range. In addition, the fabrication of resonators with ultra-small modal volumes is simple. Recently, a THz laser with an effective mode volume of 0.12(λ*/*2*n_eff_*)^3^ has been demonstrated [17].

One way to increase the selectivity of optical microsensors are adhesion layers. The resonator is covered with a thin layer, which increases the selectivity for one particular analyte [18]. The short evanescent field in the visible requires thin adhesion layers. The detection schemes works only if the resonator mode interacts with the analyte. Due to the large wavelength of THz radiation its evanescent field penetrates further outside of the resonator and eases the requirements on the thickness of the adhesion layers.

### 

#### THz Quantum-Cascade Lasers

The main problem for optical systems in the THz range are the sources. The available solutions are either bulky, e.g., gas lasers [19], or have very low output power, e.g., difference frequency generation [20]. The development of the first THz quantum-cascade laser (QCL) in 2002 changed the situation completely [21]. Now a compact and coherent source exists. The characteristics of a THz-QCL are: electrical pumping, high output power and designable gain. The active region of every QCL is based on a semiconductor heterostructure. In contrast to a regular semiconductor laser [22], where the optical gain is generated by an electron-hole recombination across the bandgap, the QCL uses only the conduction band. The grown heterostructure creates quantized energy levels for electrons. The optically active transition takes place between two of those states. The main advantage is the possibility to control all gain parameters by design. The photon energy and the dipole matrix element of the lasing transition are completely determined by bandstructure engineering or, in other words, by the thickness of the individual barriers and wells. This concept allows the realization of THz-QCLs emitting from 1.2 to 5 THz [23,24].

The THz-QCL used throughout this work is based on a gallium-arsenide (GaAs)/aluminum-gallium-arsenide (AlGaAs) superlattice and is grown by molecular beam epitaxy. The calculated bandstructure for our device is shown in Figure 1(b). It is based on the so-called resonant phonon depopulation scheme [25]. The gain maximum is designed around 2.65 THz. A thorough discussion of the active region is given in [26].

The important parameter for the detection limit is the linewidth of the resonance as it defines the minimum detectable frequency shift. THz-QCLs in continuous wave operation (CW) at a stabilized temperature show a linewidth of 0.1 MHz [27]. This is already enough to resolve the pressure broadening of gas lines [28]. An active stabilization with a frequency reference narrows the linewidth further to 6.3 kHz [27]. Phase-locking leads to a linewidth of only 1 Hz [29]. This excellent frequency stability makes THz-QCLs ideal candidates for local oscillators in heterodyne detection systems [30,31].

## 3. Active Photonic Crystal

PhCs offer unique properties for the manipulation of light on a sub-wavelength scale: the full dispersion relation can be designed. Compared to normal microdisks or microtoroids, the degree of control is much higher. The standard PhC geometry is a two dimensional array of holes in a thin membrane. These structures are compatible with standard planar processing technologies. Defects are used to realize high-Q cavities with small modal volumes. These resonators achieve quality factors in the range of 10^6^ [32].

Here, we do not use defect states of a PhC but band edges states. They are characterized by an extremely flat dispersion relation resulting in a low group velocity. These *slow light* regions can be used to confine light efficiently inside a resonator; no defects are required [33]. A strong gain enhancement is predicted due to the group velocity anomaly [34]. For the simulations we use exclusively two-dimensional models throughout this work. The real devices are embedded in metallic waveguides [35], which prevent out-of-plane scattering. Therefore, effects such as bandgap narrowing, typically present in a dielectric slab waveguide [36], can be neglected. The actual devices can be described very well using solely 2D models [37,38].

**Figure 2.**
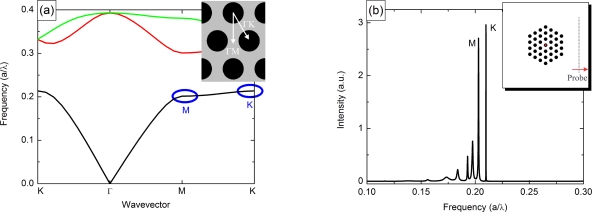
**(a)** Calculated bandstructure of the PhC for *r/a* = 0.3 and *n_pillar_* = 3.65 using the *MIT Photonic Bands* package [39,41]. The full bandgaps for TM-polarized light are clearly visible. The designed lasing points are *M* and *K* in the lowest band. **(b)** Predicted modes using finite-difference time-domain calculations [40]. The two lasing modes show a quality factor of 10^3^. The inset shows a schematic of the computational cell; the source is positioned in the central pillar, the probe at the side of the cell.

The PhC used in this work is an array of isolated pillars surrounded by air. The pillars are arranged in a hexagonal lattice, a schematic is illustrated in the inset of Figure 2(a). This type of PhC shows full bandgaps for TM-polarized light [36], the polarization emitted by QCLs. The pillars are modeled with a ratio *r/a* of 0.3, where *r* is the pillar radius and *a* the PhC period. The refractive index is set to 3.65, a measured value for GaAs in the THz regime [39]. A calculated bandstructure for the PhC used in our devices is presented in Figure 2(a). The flat regions at the *M* and the *K*-point can be used as the lasing modes. The wide, forbidden bands around the high symmetry points ensure a stable single mode emission.

The corresponding finite-difference time-domain calculations using the *MIT Electromagnetic Equation Propagation* package [40] for a perfect, loss-less structure are illustrated in Figure 2(b). The two modes at the *M* and the *K*-point are clearly visible. The simulated Q-values for the lasing modes in our device are on the order of 10^3^. This low quality factor is further reduced by losses due to the metallic waveguide to typically 10^2^ [17,35]. Such a low value is hardly tolerable for purely passive resonant microsensors. It would lead to a linewidth of 25 GHz in our case. However, the lasing linewidth is not limited by the waveguide losses. As already mentioned in Section 2, the linewidth of a THz-QCL in CW mode is on the order of 0.1 MHz [27].

**Figure 3.**
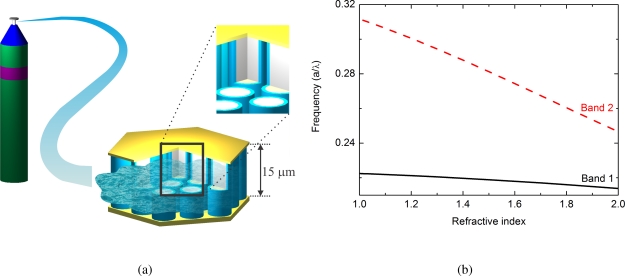
**(a)** Schematic of the active PhC sensor. The size of our resonator is comparable with passive microsensors in the visible [11]. The height is 15 *μ*m, the diameter around 200 *μ*m. **(b)** Resonance frequency shift for Band 1 and 2. Higher bands show a stronger effect of the resonance position on the refractive index.

The fabricated resonator consists of 15 *μ*m high pillars which are embedded in a metallic waveguide. The distance between the pillars is 26.6 *μ*m, the period of the PhC. The metal confines the optical mode in the vertical direction. A schematic of the PhC sensor is shown in Figure 3(a). The pillars are fabricated directly from the active THz quantum-cascade layer sequence. In this configuration, the pillars provide the required optical gain and confine the mode in lateral direction via slow light. The metallic waveguide is used to apply bias to each pillar. It is also required to achieve a vertical mode confinement as the pillar height is one order of magnitude smaller than the emission wavelength. Details about the fabrication are given in [38].

The dimensions of the structure are on the order of typical microchannels [42]. The large, open space between the individual pillars is essential for the sensor application, as it allows for a free propagation of the gas through the resonator. The injected gas changes the resonance condition of the PhC. The expected frequency variation as a function of the refractive index is illustrated in Figure 3(b). As the figure of merit we use the theoretical sensitivity *S′* = −1/*f df*/*dn*, where *f* is the frequency and *n* the refractive index. This allows us to compare our sensor to published devices across different frequency regimes. Calculation yield a *S′* of 0.025 for Band 1 [7], which is comparable to PhC gas sensors in the visible [43]. The sensitivity can be improved drastically if higher bands are used. Simulations show that Band 2 has an increased *S′* by one order of magnitude, as illustrated in Figure 3(b).

## 4. Results and Discussion

### 4.1. Gas Sensing

In this first experiment, we demonstrate an integrated optical microsensor with an embedded light source. There is no need to couple an external light source into the resonator. The information is encoded in the line position and not in an absolute intensity, making the light collection very simple. In combination with an electrical pumping of the source, we achieve a simple and robust system. Oxygen and argon are used as the unknown gases. Neither gas has any sharp absorption line around the lasing frequency. We sense only the direct change of the refractive index due to the additional gas in between the pillars. Therefore, a comparable frequency shift is expected in both cases. We perform here non-specific sensing [44,45].

The experiments are all performed under equal conditions. The lasers are operated in pulsed mode at a frequency of 30 kHz and a duty cycles of 5%. The temperature and the applied bias are kept constant during the entire measurement. The spectra are recorded using a Fourier-transform infrared spectrometer, which is purged with dry air. Due to the low operating temperature of THz-QCLs, we have to keep the samples inside a vacuum cryostat and cool them to 5 K [10]. The gas is injected using a digital injection scheme. One gas cycle corresponds 0.16 L of gas at a pressure of 10 mbar and room temperature. The volume of the cryostat is approximately 0.45 L.

**Figure 4.**
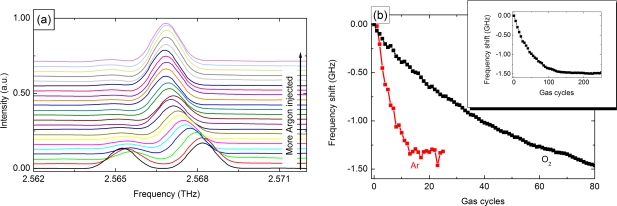
**(a)** PhC Spectrum at an applied bias of 18.4 V. There is a clear red-shift with an increasing argon concentration. The two modes visible prior to gas tuning are caused by breaking the six-fold symmetry due to processing imperfections [51]. **(b)** Line shift for argon and oxygen. Both gases show an almost linear frequency shift with increasing concentration. It saturates at 1.5 GHz or 10^−3^ times the center frequency.

The injected gas builds a thin ice film due to the low heat sink temperature of the THz-QCL. The reduction in the refractive index contrast between the pillars and the surrounding medium leads to a red-shift of the emission frequency. A series of measured spectra for argon with increasing gas concentration is presented in Figure 4(a). The line shift from its original position for argon and oxygen is illustrated in Figure 4(b). An almost linear tuning is achieved for both gases. For higher concentrations a saturation of the frequency shift appears. The lower saturation concentration of argon is an effect of its higher atomic mass, which enhances deposition to cold surfaces [46].

As neither argon nor oxygen have any sharp absorption lines close to the lasing frequency, the total frequency shift is identical and very small. In addition, the PhC used shows a weak dependence of the resonance frequency on the surrounding medium. Therefore, the detuning for both gases is limited to 1.5 GHz or 10^−3^ times the central lasing frequency. This total shift is comparable to PhC tuning experiments in the visible by xenon or nitrogen sublimation [47]. Microsensor in the near-infrared show similar values for the frequency shift of the cavity resonance [5,48–50].

### 4.2. Discussion and Outlook

Before this sensor can leave the lab and be used in every day life, it is necessary to improve its performance. The most obvious drawback is the low operation temperature of THz-QCL. Especially the use of a cryostat limits the applicability. New electron transport schemes could already improve the maximum lasing temperature of THz-QCLs significantly [10]. The modeling of the active region based on non-equilibrium Green’s functions [52,53] or Monte-Carlo simulations [54,55] shows an excellent agreement with the experiment. Based on this simulations new active regions are proposed, which are capable of room temperature operation [56,57].

The sensitivity of the detector can be improved in a much simpler way. The first device is realized in the lowest PhC band. There, the optical mode shows the strongest overlap with the pillars which favors the laser realization. Higher bands show a stronger dependence of the resonance condition as a function of the refractive index. Changing the operation from Band 1 to Band 2 in the PhC bandstructure increases the sensitivity already by one order of magnitude. However, the energy confinement is reduced in higher bands.

**Figure 5.**
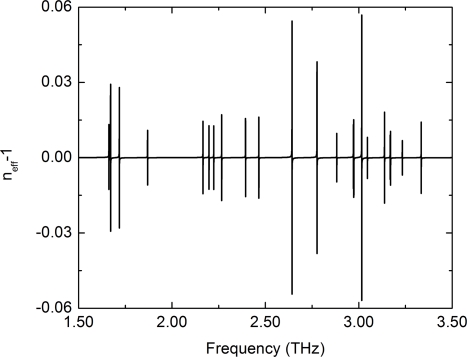
Refractive index of water vapor at 10 mbar and 10^−6^ mol [16]. The individual water absorption lines lead to a strong dispersion.

The bid advantage of this sensor concept is its simplicity and potential selectivity. It is an electrically pumped, active sensor. There is no need to couple light into a high-Q cavity. The information is encoded in the line position and not in an absolute intensity. The frequency change happens instantly with variations of the gas concentration and is fully reversible. Due to the realization in the THz frequency region, it is possible to make full use of the chemical fingerprint of different molecules. The emission frequency of the bare laser is defined purely by geometry [38]. Therefore, it is easy to design it close to an absorption line. This sharp absorptions lead to an noticeable index change only in a narrow frequency range. Far away from such a resonance, the refractive index is unchanged. The sensor can be sensitive to only one molecule within a mixture of unknown gases. The refractive index of water vapor at 10 mbar is presented in Figure 5 as an example.

The narrow linewidth of THz-QCLs leads to an extremely low detection limit. A THz-QCL with an emission frequency of 2.5 THz and an unstabilized linewidth of 0.1 MHz correspond to a passive resonator with a quality factor of 2.5 × 10^7^. If we assume the same unstabilized linewidth, we achieve theoretically a minimum detectable index change for the device described of 1.6 × 10^−5^ RIU (refractive index unit).

## 5. Conclusions

We have realized a gas sensor operating in the THz spectral region. The device is based on an active PhC which is fabricated directly from the gain region of a THz-QCL. Thereby, the PhC can be pumped electrically, leading to laser emission. There is no need to couple an external source into the cavity. An injected gas shifts the resonance frequency of the PhC and thereby the emission frequency of the laser. This first experiment shows the possibility of non-specific gas sensing with oxygen and argon as the two unknown gases. A maximum frequency shift of 1.5 GHz, corresponding to 10^−3^ of the center lasing frequency, has been measured. The device is operated in the lowest PhC band, which gives a theoretical sensitivity *S′* of 0.025. In the next step, the operating point of the active PhC will be shifted to higher bands. Thereby, the sensitivity is increased by one order of magnitude.
